# The Vascular-Immune Hypothesis of Alzheimer’s Disease

**DOI:** 10.3390/biomedicines11020408

**Published:** 2023-01-30

**Authors:** Rashi I. Mehta, Rupal I. Mehta

**Affiliations:** 1 Department of Neuroradiology, Rockefeller Neuroscience Institute, West Virginia University, Morgantown, WV 26506, USA; 2 Department of Neuroscience, Rockefeller Neuroscience Institute, West Virginia University, Morgantown, WV 26506, USA; 3 Rush Alzheimer’s Disease Center, Rush University Medical Center, Chicago, IL 60612, USA; 4 Department of Pathology, Rush University Medical Center, Chicago, IL 60612, USA

**Keywords:** Alzheimer’s disease, beta-amyloid (βA), glymphatic–lymphatic system, mixed pathologies, neurovascular unit (NVU), perivascular unit (PVU), tau

## Abstract

Alzheimer’s disease (AD) is a devastating and irreversible neurodegenerative disorder with unknown etiology. While its cause is unclear, a number of theories have been proposed to explain the pathogenesis of AD. In large part, these have centered around potential causes for intracerebral accumulation of beta-amyloid (βA) and tau aggregates. Yet, persons with AD dementia often exhibit autopsy evidence of mixed brain pathologies including a myriad of vascular changes, vascular brain injuries, complex brain inflammation, and mixed protein inclusions in addition to hallmark neuropathologic lesions of AD, namely insoluble βA plaques and neurofibrillary tangles (NFTs). Epidemiological data demonstrate that overlapping lesions diminish the βA plaque and NFT threshold necessary to precipitate clinical dementia. Moreover, a subset of persons who exhibit AD pathology remain resilient to disease while other persons with clinically-defined AD dementia do not exhibit AD-defining neuropathologic lesions. It is increasingly recognized that AD is a pathologically heterogeneous and biologically multifactorial disease with uncharacterized biologic phenomena involved in its genesis and progression. Here, we review the literature with regard to neuropathologic criteria and incipient AD changes, and discuss converging concepts regarding vascular and immune factors in AD.

## 1. Introduction

Alzheimer’s disease (AD) is a chronic neuropathological condition that manifests from a host of biological factors that collectively cause damage and loss of neurons, synapses, and supporting cells of the brain (i.e., glia), ultimately manifesting with brain atrophy [1]. Clinically, AD dementia features memory and executive function deficits, with subsequent progressive, global cognitive decline [1,2]. Overall, AD is the sixth leading cause of death in the United States. Approximately 6 million Americans are currently afflicted with AD dementia and in the absence of effective therapies, this figure is expected to expand to approximately 14 million by 2050 [2].

Early-onset AD (EOAD) occurs before the age of 65 years and is often associated with autosomal dominant inheritance, whereas late-onset AD (LOAD) manifests after age 65 years and does not typically exhibit Mendelian transmission [3]. EOAD is caused by highly penetrant mutations in amyloid precursor protein (APP), presenilin 1 (PSEN1), or presenilin 2 (PSEN2) genes, while nonfamilial cases may involve trisomy of chromosome 21 that harbors the APP gene [4,5,6]. These genetic aberrations influence brain levels and quality of beta-amyloid (βA) peptide, the primary constituent of βA plaques. Apolipoprotein E (ApoE) is linked with LOAD and distinct ApoE isoforms have been shown to divergently regulate βA production and aggregation [7]. Both EOAD and LOAD are characterized by preclinical and prodromal, i.e., mild cognitive impairment (MCI), stages that together span decades [2].

Despite knowledge on genetic associations and incipient phases of disease, the precise etiology of AD remains uncertain [8]. Investigation is complicated by the fact that many patients with AD also harbor comorbid diseases [9]. Nevertheless, a panoply of explanations has been put forth regarding the potential causes of this disease [10]. These hypotheses have been a driving force for clinical trials, yet disease-modifying treatments remain limited. Given the observation of widespread βA aggregation in diseased brains, βA has generally been regarded as the primary pharmacological target for AD [11] while other targets and theories continue to be debated. Here, we review the neuropathology of AD, early and incipient brain changes, and evidence in support of a vascular–immune basis of disease genesis. Furthermore, we summarize elements of the neurovascular and perivascular unit, which are essential to brain health and metabolism, and describe knowledge gaps and emerging directions in the field.

### 1.1. Neuropathologic Criteria for AD

Alzheimer’s disease neuropathologic change (AD-NC) encompasses extracellular βA deposits and intraneuronal tau protein inclusions. Spatiotemporal propagation of these lesions generally follows stereotyped patterns in the brain, as summarized in National Institute on Aging–Alzheimer’s Association (NIA-AA) guidelines [12,13]. 

βA derives from the amyloid precursor protein (APP) [14] and forms intracerebral senile plaques (SP) that are morphologically heterogeneous and graded according to the Thal scheme [13]. βA is loosely arranged in diffuse plaques (DP), diffuse amyloid lakes and subpial bands. When harboring thick neurites, SPs are called neuritic plaques (NP) [15]; these are separately graded by CERAD scheme [13]. Another NP subtype, the dense-core plaque, displays a central compact βA concretion that is surrounded by a loose zone of diffuse βA deposit [15]. In EOAD, βA plaques are also often arranged in so-called cotton wool plaques that are large, wispy, spherical structures that lack central βA concretions. Per current neuropathologic scoring schemes, neocortical NP densities are semiquantitatively graded [13]. The initial stage of βA deposition involves neocortical brain regions (stage I), but with disease progression, there is involvement of allocortical brain (stage II), subcortical nuclei (stage III), medulla oblongata and midbrain (stage IV) and, eventually, pons and cerebellum (stage V) [13].

In contrast to βA, tau derives from the MAPT gene (chromosome 17). Tau protein is normally found throughout the nervous system and is primarily expressed by neurons. Though its role is not fully elucidated, it is thought to have a role, in part, in neuronal microtubule stabilization. When abnormally hyperphosphorylated, tau is prone to aggregate as intraneuronal paired helical filament inclusions. Within neuronal soma, hyperphosphorylated tau protein forms neurofibrillary tangles (NFT), whereas in distal neuronal processes (i.e., axons and dendrites) it forms neurites and neuropil threads (NT). NFTs primarily localize to limbic regions during early or mild course of disease, but with disease progression the NFTs also aggregate in association cortices and subcortical gray matter nuclei, in a pattern that is distinct from that of βA. A consensus guideline suggests the Braak topographical scheme for NFT staging: stage 0 corresponds to an absence of NFT; stage I/II corresponds to NFT accumulation in transentorhinal regions; stage III/IV corresponds to NFT accumulation within limbic regions; stage V/VI corresponds to wider dispersal of NFT throughout neocortical brain regions [13]. 

### 1.2. Theories on AD Pathogenesis

While the causes of AD-NC are unproven, age is an important risk factor and a number of theories have been posited to explain the gradual age-associated accumulation of βA, NFT and NT inclusions. The **cholinergic hypothesis** proposes that AD symptoms arise due to deficiency of intracerebral acetylcholine [10]. This seminal hypothesis evolved from knowledge that the acetylcholine neurotransmitter and cholinergic neuron loss in limbic and cerebral cortical regions are fundamental features of disease. The **amyloid (or amyloid cascade) hypothesis** theorizes that aberrant βA peptides produced by sequential cleavage of APP by APP-cleavage enzymes (i.e., BACE1 and β/γ-secretases) aggregate into oligomers and insoluble extracellular plaques that damage brain neurons and lead to a range of detrimental secondary phenomena [10,16,17]. The **tau propagation hypothesis** proposes that development and deposition of aberrant hyperphosphorylated tau protein is the initiating pathological event in AD [18], whereas the **inflammation hypothesis** emphasizes the importance of activated microglia and astrocytes in disease genesis [10,19]. Meanwhile, the **oxidative stress hypothesis** asserts that a variety of conditions that cause free radical generation lead to peroxidation of membrane polyunsaturated fatty acids, thus precipitating AD lesions [10]. Similarly, the **mitochondrial hypothesis** proposes that mitochondrial dysfunction occurs upstream of the above phenomena, influencing expression and processing of APP and thereby causing aberrant βA accumulation and an associated cascade of secondary brain changes [10]. The **infectious hypothesis** proposes that a pathogen, i.e., virus, bacterium or prion, underpins disease while the **calcium homeostasis hypothesis** and **metal ion hypothesis** conjecture that calcium or metal ion dysregulation, respectively, are the initial culprit [10]. The **ion channel** [10], **cell cycle** [20], **autoimmune** [21], **epigenetic** [22] and **granuloma hypotheses** [23], among others, have also been propositioned [19]. Altogether, the diversity and number of diverging theories for AD highlight the complexity of this neurological condition and the magnitude of uncertainty regarding initial pathogenetic events. 

### 1.3. Therapeutics and Evidence in Support of Current Theories

Over the course of a century, only seven medications have been formally approved by the Food and Drug Administration (FDA) for AD treatment [11]. These include donepezil (Aricept^®^), galantamine (Razadyne^®^), rivastigmine (Exelon^®^), and tacrine (Cognex^®^), which are acetylcholinesterase inhibitors (the first drug in this class was first approved in 1996) [14]; memantine (Namenda^®^), a N-methyl-D-aspartate receptor (NMDA) receptor antagonist that was approved by the FDA in 2013; aducanumab (Aduhelm^®^), an anti-βA monoclonal IgG1 antibody that was approved in 2021; lecanemab (Leqembi^TM^), a monoclonal anti-βA IgG1 antibody that selectively targets soluble aggregated βA species, that was just approved this month [24]. Both acetylcholinesterase inhibitors and NMDA receptor antagonists are regarded as supportive therapies, only, whereas anti-βA agents have overall shown mixed results with the overwhelming majority of drugs in this class failing to show cognitive improvement in humans [25]. 

Given that genetic, pathological, and functional evidence in humans points toward imbalanced βA production and removal as potential causes for early βA aggregation in various brain regions, βA peptides have generally been considered the primary pharmacological target for AD [10]. Consequently, the amyloid hypothesis has been the prevailing theory and has driven AD drug development strategies for over a quarter century. Initially, βA species in the form of soluble oligomers, intraneuronal aggregates, and insoluble plaques were suggested to be neurotoxic in AD subjects. Data show that βA deposits appear decades prior to dementia symptoms in persons with EOAD and LOAD [25,26]. Yet, clinical trials involving immune-mediated βA removal have been met with limited success [25,27]. Aducanumab, which targets the aggregated plaque form of βA, was approved for use in persons with mild AD symptoms and showed reduction of βA plaque burden but did not translate to significant cognitive improvement. This, too, was observed in only one large-scale trial that subjected patients to prolonged duration of therapy [28]. Meanwhile, high rates of amyloid-related imaging abnormalities (ARIA; up to 43% rate of edema or effusions (ARIA-E) among ApoE Ɛ4 carriers and a 20% rate of microhemorrhages (ARIA-H) in the EMERGE high-dose group) were noted, whereas chronic impairment was documented in 1–2% of patients and therapy-related death was suspected in at least one subject [29,30]. Due to concerns regarding its risk–benefit profile, aducanumab was ultimately discontinued. Likewise, hundreds of prior candidate drugs targeting βA, including BACE1 and β/γ-secretase inhibitors have been ineffective and associated with adverse effects [16,31]. While the trial failures may be attributable to various factors, the failure of innumerable anti-βA drugs has raised questions and doubts regarding presumed roles of βA in AD [17]. 

More recently, lecanemab has shown efficacy in slowing cognitive decline in early-stage AD subjects enrolled in a large, phase III clinical trial [32,33]. While lecanemab elicited brain swelling and effusions (ARIA-E) in a subset of persons, this drug was approved under FDA’s accelerated approval pathway, based on phase II trials that incorporated 18F florbetapir positron emission tomography (PET) imaging of βA as a surrogate endpoint. Additional clinical treatment effects of lecanemab are yet to be determined and a number of other anti-βA drugs remain under investigation [34]. 

## 2. Discordance between Neuropathologic Grading Schemes and Theories of AD

Despite the requirement of βA plaques and NFTs for diagnosis of AD-NC and the use of βA as a surrogate endpoint of disease, these markers are of limited utility in disease grading. For instance, precise form(s) and toxic βA species are ambiguous and associations of DP density with cognitive impairment are weak. On the other hand, the abundance and distribution of NFTs and NPs are thought to be predictors for cognitive impairment [13,35]. Yet, NFTs and NPs are also commonly observed in individuals who are cognitively intact [35]. An investigation into risk factors associated with rapid clinical progression, from MCI to dementia, highlights the significance of cerebrospinal fluid (CSF) total tau and phosphorylated tau, but not βA^1–42^ levels, as pertinent to disease [36]. Moreover, abnormal tau protein inclusions also arise in association with other neurodegenerative conditions (i.e., tauopathies), including Pick disease, progressive supranuclear palsy, corticobasal degeneration, chronic traumatic encephalopathy, aging-related tau astrogliopathy, primary age-related tauopathy, and some forms of frontotemporal dementia [1]. Thus, NFTs and NPs are nonspecific lesions that do not accurately predict elderly persons with AD dementia [37,38]. Meanwhile, a number of alternate brain changes and protein inclusions not encompassed by AD-NC criteria are commonly associated with AD. While AD-NC, vascular diseases, and brain vascular injuries are recognized as the most common neuropathologic lesions associated with AD dementia, these entities also often overlap [39]. For a variety of reasons, it has increasingly been thought that βA is associated with, but not causative of AD. It is also increasingly accepted that significant subsets of individuals with AD dementia exhibit heterogeneous tissue substrates and unique combinations of tissue lesions [40]. As chronic systemic and neuropathologic diseases propagate in the oldest old (i.e., persons > 90 years of age), the relationship between AD dementia and established AD-NC criteria diminishes further relative to younger persons, even after correcting for mixed pathologies, suggesting that heterogeneous unknown biological factors apart from βA, NFTs, and other recognized neuropathologies are determinants of the disease [41,42,43].

### Shifting Perspectives: Inconsistencies of the Amyloid Cascade Hypothesis

Despite intensive investigation, direct mechanistic links between βA and AD dementia remain unproven and βA has never been universally accepted as the underlying cause for AD. Over the past decade, a number of studies have suggested that βA toxicity is dependent on the presence of phosphorylated tau inclusions [18]. Moreover, the ApoE genotype has been recognized to modify the risk of AD and cognitive decline through both βA-dependent and βA-independent mechanisms [7]. Increasing evidence highlighting discrepancies between AD-NC and established diagnostic criteria for AD dementia has led to reappraisals of the amyloid hypothesis. A revised amyloid hypothesis suggests that lower-order soluble βA polymers that are imperceptible on histology are the true neurotoxic species in AD [17]. Some researchers maintain that βA and NFTs co-occur, but suggest that disease progression is mediated by tau pathology [18]. Several investigators now believe that tau pathology is an integral substrate of LOAD and propose that tau toxicity is in some way triggered by βA or βA-related biology. Recent data also uncovers marked variability of tau strains and conformations, and suggests that selective tau recognition by chaperones may differentially influence the accumulation and effects of proteotoxic tau species [38]. Though tau pathology is largely characterized according to NFT burden, research also highlights a diversity of post-translational tau modifications prior to NFT formation [44]. As with βA, soluble tau forms are increasingly thought to represent the true neurotoxic species. Some recent data also suggest that AD pathogenesis occurs due to the effects of long-term elevation of βA concentration, rather than by transient overproduction or impaired clearance [45]. Other studies stress aberrant protein aggregation properties [46]. Still, epidemiological evidence implies that other phenomena, lesions and/or brain “hits” are contributory [41,47,48] and highlight perplexing questions about AD: why are some mutation carriers from AD kindred resistant to βA plaques and NFTs [48]? Why does cerebral amyloid angiopathy (CAA) variably co-occur with AD-NC? Why are non-βA and non-tau protein inclusions observed in heterogeneous combination with disease [40]? Why are vascular pathologies so frequent in association with disease [41,42,43]? Why do SuperAgers exhibit AD-NC, but no clinical evidence of disease [1,40]? In light of recent literature, these inconsistencies emphasize the need for distinctions between diagnostic biomarkers and molecular targets for AD therapies [14].

## 3. New Theories: Convergence on Vascular and Neuroimmune Homeostatic Factors

Over the past two decades, investigation into alternative and early-stage AD biomarkers has underscored potential new AD targets [17,49]. Given the convergence of a number of early biological phenomena around cerebral vessels, it is now clear that cerebrovascular contributions to AD have been underrecognized. Increasing data show that inflammatory changes and loss of vascular wall integrity are the earliest identifiable lesions in persons with AD, preceding βA and tau accumulation [50,51]. Interestingly, βA has also been reported to have cytokine-like immune functions, to act as an early responder to diverse stress stimuli, and to demonstrate antimicrobial properties that have not been fully explored [52,53,54,55,56]. 

In parallel, improvements in microscopy and imaging technologies have led to revised concepts in neuroanatomy, neuroimmunology, and brain barrier biology. The meningeal lymphatic system has emerged as a critical player in neurophysiology [57,58,59]. This new literature challenges long-held assumptions regarding the brain’s “immune privilege”, as it reveals dynamic functional cross-talk between the brain and peripheral cells and tissues through various routes and a myriad of cellular and molecular mechanisms [60,61]. Moreover, studies into intracerebral fluid physiology in mammalian models have demonstrated that a brain-wide fluid system, generally referred to by some as the glymphatic system, transports solutes and waste metabolites through the cerebrum by way of interstitial and perivascular spaces (PVS) [62,63,64]. When the glymphatic and lymphatic systems are dysfunctional, CSF tracer and solute transport is impeded [62,65]. Furthermore, this brain drainage system links the health of cerebral vasculature with brain inflammation, stressing critical inter-relationships between brain blood vessels and neuroimmunity. Given these recent neuroscientific advances and knowledge on lymphatic diseases in peripheral tissues, a vascular–immune basis for AD is conceivable and evidence in support of specific pathways and mechanisms of brain fluid and metabolite clearance is now emerging rapidly across species.

### 3.1. Brain Border Macrophages in AD

Pivotal roles of macrophages at brain surfaces and their significance in brain homeostasis are highlighted in recent literature [66,67,68,69,70]. Within the cranium, but outside brain parenchyma, macrophages known as central nervous system (CNS)-associated macrophages (CAMs) or parenchymal border macrophages (PBMs) reside within meninges, PVS, and choroid plexus and are involved in regulating essential exchange between CNS parenchyma and the periphery. These macrophages are thought to support and maintain brain barrier properties, control drainage of CNS antigens, and aid in clearance of waste metabolites [66,67]. Moreover, macrophage populations at brain borders are shown to be phenotypically diverse [66,68]. Due to their strategical positioning as well as roles in CSF processing and coordination of reciprocal cross-talk with other cell types, CAMs/PBMs act as critical regulators of brain metabolism and gatekeepers of general neuroimmune processing [67,68]. Evidence suggests that they control the environment and tune entry of immune cells from the CSF and blood into the brain parenchyma and, in turn, regulate the exchange of diverse molecules between the bloodstream, periphery, and brain [67]. PVS macrophages are increasingly thought to have underrecognized roles in CAA and AD [68,69]. In a transgenic TgCRND8 AD mouse model, their depletion by clodronate liposome resulted in a prominent increase in PVS βA^1–42^ deposits [70]. Moreover, CAA pathology was mitigated in this model by chitin-mediated PVS macrophage enrichment [70]. Separate works demonstrated that PVS macrophage density is associated with arterial motion, brain extracellular matrix components, CSF flow dynamics, and clearance of proteins [68,69]. Moreover, the number of PVS macrophages and efficiency of phagocyte activity was shown to be highly associated with perivascular βA deposits [69]. Collectively, these studies suggest that activity and manipulation of PVS macrophages could be integral in the regulation of CAA and AD. 

### 3.2. Peripheral Monocytes and Macrophage Infiltration in AD

While microglia are the principal innate immune cell of the brain and originate from yolk sac erythromyeloid progenitor cells from which they migrate, propagate, and spread during embyogenesis, it has been shown that peripheral monocytes are continuously replenished in adult brains [71,72]. This includes healthy adult brains, in which the recruited cells engraft and differentiate into parenchymal microglia, i.e., new brain resident cells, or remain a distinct population [73,74,75]. While this recruitment occurs in limited numbers in healthy subjects, experimental AD subjects exhibit accelerated recruitment of monocytes from the periphery [73,74,75]. Invading monocytes and macrophages that engraft the brain have been shown to derive from circulating Ly-6C^hi^ monocytes or from bone marrow-derived progenitors such as granulocyte–macrophage progenitors (GMPs), or other hematopoietic stem cell progeny [66,73,74]. In a study that employed a green fluorescence protein (GFP)-bone marrow chimeric APP transgenic mouse model (APP23 mice) to study CNS invasion by hematopoietic cells, it was discovered in nontransgenic control mice that the majority of recruited GFP-positive cells localized to brain borders (i.e., PVS, meninges, choroid plexus, and ventriclular ependyma) [75]. In contrast, in βA-depositing APP23 mice that develop CAA, a significant portion of recruited GFP-positive cells localized to brain parenchyma, particularly to neocortical areas that exhibit high βA load [75]. These data illustrate critical differences in peripheral myeloid cell recruitment patterns between AD and non-AD subjects, suggesting a role in disease evolution and implying that their targeting may modulate disease course. 

### 3.3. Microgliosis and Other Vascular-Immune Factors in AD

Reactive microglia, i.e., resident brain phagocytes, invariably accompany AD-NC [76,77,78,79,80,81,82,83]. In fact, activated microglia and βA deposits spatially overlap in cerebral cortices of subjects with MCI [77,82], while baseline brain microglial activation and activated hippocampal microglia are significantly increased in subjects with AD [71]. In response to the accrual of βA plaques, adjacent microglia increase their expression of CD11b, CD68, and complement receptor 3 [82,84]. Moreover, in vitro analyses demonstrate the ability of βA to stimulate pro-inflammatory cytokine (including interleukin (IL)-1β, IL-6, IL-8, tumor necrosis factor (TNF)), chemokine, and neurotoxin production by human microglia, causing neuronal damage [85,86,87]. In response to βA, microglia also secrete proteolytic enzymes and express receptors to promote βA phagocytosis and clearance. This includes the upregulation of class A scavenger receptors (SR-A), the receptor for advanced glycation end-products (RAGE), CD36, toll-like receptors (TLRs) and b1 integrins [82,88]. Therefore, βA may signal in combination with a host of immune factors to facilitate the recruitment of peripheral immune cells into brains of persons with AD, which may impact on brain function [75]. Some data also suggest that microglia form a protective barrier around βA plaques, compacting βA fibrils into tightly packed and less toxic forms [71,80].

TREM2 (triggering receptor expressed in myeloid cells 2) has also emerged as a key player in microglial and AD biology [71,89,90]. This protein has been shown to function as a receptor for βA and has affinity for its oligomeric forms [89]. The TREM2 cell surface receptor promotes microglial association with βA plaques and via its interaction with the activating adaptor protein DAP12, it plays critical roles in chemotaxis, survival, and proliferation of myeloid cell populations and phagocytosis of a variety of substrates, including apoptotic neurons, bacteria, low-density lipoprotein (LDL) and other lipoproteins, and βA [71]. Loss of TREM2 function diminishes microglial responses to βA plaques, enabling a more toxic state [90]. Rare TREM2 variants are now known to increase the risk of LOAD by two- to four-fold [78].

Variances in analytes from TNF, complement, and coagulation pathways are also demonstrated in persons with MCI [79]. These data demonstrate that neuroinflammatory sequelae manifest early in the course of AD and suggest that they may represent cardinal features of disease. In line with this, plaque-independent inflammation has been demonstrated in neurons that harbor increased levels of soluble and oligomeric βA [83]. Cross-sectional studies further support the hypothesis that neuroinflammation may influence AD pathology at early stages of disease [79,91,92], while epidemiological evidence from several studies highlight decreased prevalence of AD and delayed disease progression in populations treated with long-term anti-inflammatory therapies [93,94]. 

Adding to this evidence, a majority of recently identified AD risk genes discovered on genome wide association studies such as ABCA7, BIN1, CD33, CR1, INPP5D, MS4A6A, PLCG2SHIP1, and TREM2 are highly or exclusively expressed by macrophages and microglia [71,76,77,78,79]. Additional risk genes such as SORL1, CD2AP, PICALM, PTK2B involve sorting receptors, cell membranes, and lipid metabolism [77,95]. These findings underscore the potential critical roles of myeloid cells, intracranial transport, and transport molecules in AD pathogenesis [80,81,82,83,84,85,86,87,88,89,90,91,92,93,94,95]. 

### 3.4. Imaging Evidence of Early Hypometabolism and Vascular and Perivascular Dysfunction

In support of a vascular–immune theory of AD, clinical imaging evidence demonstrates that subtle, asymptomatic vascular changes and blood flow aberrations precede βA and NFT formation, cerebral cortical atrophy, and cognitive decline [51,96,97,98]. 18F-FDG-PET imaging studies document decreased glucose uptake in specific brain regions during prodromal stages of AD [99]. As abnormal FDG uptake is an indicator of resting-state brain glucose hypometabolism, this represents a biomarker of brain vascular dysfunction. Longitudinal 18F-FDG-PET studies have shown that reduced hippocampal FDG uptake predicts cognitive decline with high sensitivity and specificity years in advance of clinical AD symptoms [100]. Furthermore, high resolution dynamic contrast-enhanced (DCE) MRI analyses demonstrate blood–brain barrier (BBB) injury in the hippocampi of individuals with MCI, suggesting vascular tunica intima damage as an early independent biomarker of cognitive impairment [50,51]. DCE MRI analyses also suggest that BBB breakdown contributes to ApoE4-associated cognitive decline, independent of AD-NC [98]. 

Enlarged PVS have also emerged as an early imaging biomarker of dementia and AD. While the physiology of these fluid channels remains an area of intensive investigation, it is clear that PVS play underrecognized roles in interstitial fluid and metabolite homeostasis [68,69,101]. PVS surround intracerebral and meningeal vessels and, as described above, provide a unique niche for CAMs/PBMs which are intricately involved in brain health and PVS function [68,69,102]. PVS structures enlarge with age, BBB permeability, hypertension, CAA and other small vessel diseases [101,102,103], though the causes and effects of this phenomenon are unclear. Various PVS metrics, including number, diameter, and volume, may be quantified in vivo on structural MRI, though further investigations are needed to optimize and harmonize MRI quantitation techniques [103,104,105].

### 3.5. Cardiac and Cardiovascular Disease Effects in AD

Clinicopathologic investigations document a variety of cardiac and extracranial and intracranial vascular changes and pathologies in persons with AD [1,106,107,108,109,110,111,112,113,114]. Cardiac and extracranial cardiovascular diseases associated with AD include structural or functional heart diseases such arrhythmic disorders and coronary artery, valvular, and hypertensive diseases [1,112]. Associated extracranial carotid artery changes include stenosis, occlusion, and emboli, whereas intracranial cerebral large vessel pathologies include circle of Willis atherosclerosis, vasculitis and vasculopathies [1,112]. Intracerebral small vessel diseases encompass a multitude of processes, most notably CAA and arteriolosclerosis [1,112]. 

Microscopic and macroscopic infarcts, i.e., secondary effects of cardiovascular diseases, are predictive of brain neuron and axon density as well as AD dementia risk [1,106]. Longitudinal community-based studies confirm that macroscopic and microscopic infarcts are independent predictors for cognitive decline, though pathophysiological mechanisms are not fully defined [104,107,108]. Other cardiovascular risk factors such as atrial fibrillation and congestive heart failure are also linked to frequency of AD-NC [105,106,108]. 

CAA is characterized by the deposition of βA within the tunica media and tunica adventitia of cerebral arteries, arterioles, capillaries, and rarely venules. CAA preferentially involves deposition of βA^1–40^ species and may be associated with microhemorrhages or overt, lobar hemorrhages [1,109]. Clinicopathological studies demonstrate significant associations between moderate-to-severe CAA with age and ischemic pathologies [115]. Community-based studies notably document an association between CAA and cognitive impairment, even in the absence of AD-NC [115]. Data show that CAA, arteriolosclerosis, and atherosclerosis severity independently predict increased odds of AD, even after adjusting for NPs, NFTs, and other common age-related neuropathologies [41,114,116]. 

Interestingly, recent studies also document associations between watershed brain pathology and hypoperfusion injury, and NFTs [106,117]. Mechanisms of brain cell damage and loss associated with vascular diseases are diverse, and may include apoptotic, oncotic, and mixed cell death pathways; however, pathogenetic mechanisms associated with vascular diseases are not fully elucidated. In both large vessel diseases and small vessel diseases, endothelial dysfunction, oxidative stress, and inflammation may contribute to vascular-mediated injuries. A study that employed a time-varying effect model showed that while some vascular diseases (i.e., atherosclerosis and CAA) are associated with a lower level of cognition, their detrimental effects are relatively stable over time, whereas others (e.g., macroscopic infarcts) are associated with progressive deleterious effect on cognition [42]. On the other hand, microinfarcts and arteriolosclerosis were not associated with temporal cognitive changes. These data indicate that age-related vascular pathologies are differentially related to cognitive trajectories over the lifespan [42]. Due to several complexities, no formal criteria exist currently for pathological staging of vascular diseases in AD. 

## 4. Potential Roles of the Glymphatic–Lymphatic System in AD

Since the re-discovery of pseudolymphatic [62,117] and lymphatic [118,119] channels in mice, glymphatic and lymphatic transport mechanisms have been increasingly characterized as highly organized fluid transport systems, though investigations in humans are limited [63,101]. Literature on glymphatic system demonstrates that perivascular channels lined by aquaporin 4, i.e., water channels on the end-feet of perivascular astrocytes, provide a major driving force for fluid flow across the brain while the meningeal lymphatic system effluxes intracranial fluid to cervical lymph nodes [58,59], thus promoting elimination of soluble proteins and metabolites from brain parenchyma [63]. Although the concept of lymphatic-mediated βA and metabolite clearance in humans is not novel [117,118], specific anatomical routes and mechanisms for fluid flow in human brain have been more clearly defined in recent years [60,64,69,120,121,122,123,124]. While aspects of intracerebral fluid dynamics remain controversial, knowledge on intraparenchymal and peri-parenchymal fluid pathways revises concepts of brain physiology, in that interstitial fluid (ISF) and CSF are increasingly accepted to function as the brain’s lymphatic fluid [62,120,121,122]. The glymphatic and lymphatic systems, originally characterized as separate parallel systems involved in intracerebral [62] and extracerebral [58,59] fluid transport, respectively, are now understood to be anatomically coupled [60] and functionally linked [61]. Furthermore, extracellular βA and tau clearance are significantly hampered in the rodent brain in the absence of a functionally draining dural lymphatic system [57]. Altogether, these data suggest the need for re-examination of mechanisms of macromolecule and metabolite clearance, brain homeostasis, and intracranial fluid regulation in humans. Although the role of βA in the context of AD pathogenesis is unclear, perivascular βA deposition patterns notably recapitulate intracerebral fluid inflow and outflow paths at brain borders, suggesting that intracerebral βA accumulation may occur as a result of flow or clearance failure or, alternatively, may cause glymphatic–lymphatic impedance [69]. Thus, more detailed histological, physiological, and epidemiological investigations of βA risks and deposition patterns along the brain and cerebrovasculature in the context of CAA and AD are needed. 

### 4.1. Clearance Mechanisms at the Neurovascular-Perivascular Interface

**Figure 1:**
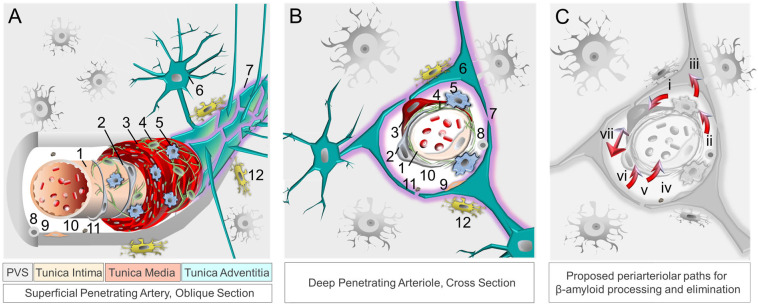
The neurovascular–perivascular unit and recognized βA and brain metabolite clearance mechanisms. Neurovascular and perivascular elements facilitate nutrient exchange as well as metabolism and removal of metabolic wastes, including βA. The neurovascular unit, depicted in oblique section (**A**) and cross section (**B**), is composed of (1) endothelial cells, (2) pericytes, (3) smooth muscle cells, (4) reticular cells, (5) macrophages, and (6) astrocytes comprised of (7) aquaporin-4 expressing astrocytic end-feet. The perivascular space harbors (8) other immune cells, (9) fibroblasts, (10) apolipoprotein E, (11) and extracellular matrix components, while (12) microglia are present in adjacent brain tissue. Metabolites, including βA and tau, are ultimately cleared from the brain by interstitial fluid flow in combination with other mechanisms (**C**), including phagocytosis [by (i) vascular smooth muscle cells, (ii) macrophages, (iii) glial cells, and (vi) pericytes]; (iv) apolipoprotein E-mediated processes); (v) transcytosis across the blood–brain barrier; receptor-mediated endocytosis (involving various immune and other cell types); (vii) perivascular glymphatic mechanisms [62,63,121]. Abbreviation: β, beta; PVS, perivascular space.

**Figure 2:**
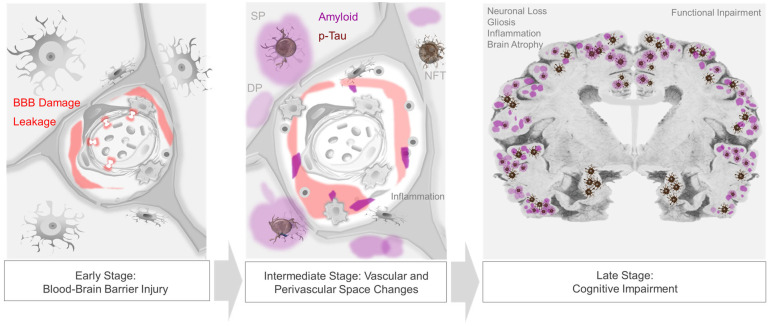
Progression of neurovascular–perivascular and parenchymal brain changes in AD. Blood–brain barrier (BBB) damage and infiltration of serum and blood cellular elements occur in prodromal, i.e., early-stage AD (**left panel**). Vascular and/or inflammatory insults may further induce transudates in perivascular spaces, impede interstitial fluid drainage, and cause impaired nutrient exchange, edema, cytokine and metabolic waste accumulation, and reactive inflammation and dysregulation (**middle panel**). In later stages of disease, extracellular βA plaques and intraneuronal neurofibrillary tangles, as well as other protein inclusions, are frequent and are associated with progressive cognitive impairment (**right panel**).

Aside from permitting nutrient transfer into brain, the neurovascular–perivascular unit (NVU/PVU) is a critical brain border and vital interface responsible for the transfer of metabolic wastes, immune cells, immune signals, and other small molecules into and out of brain [102,104]. Within the brain, the NVU is comprised of endothelial cells, pericytes, smooth muscle cells, reticular cells, macrophages, and astrocytes, among other cell types, whereas PVUs are situated along the perimeter of intracerebral blood vasculature (Figure 1). Though cellular and molecular mechanisms involving the NVU/PVU remain under investigation, knowledge on this brain barrier is evolving swiftly and available data suggest that anatomic, physiologic and pathologic changes at this interface are essential to brain homeostasis [69,104]. Brain metabolites, including βA, are cleared from the brain by ISF, CSF and blood [125]; through phagocytosis by pericytes, vascular smooth muscle cells, macrophages, and glial cells; through transcytotic processes across the BBB; and through receptor-mediated endocytosis involving various cell types [14,126,127] (Figure 2). Detection of tau and other brain proteins within blood implies that they are also cleared from the brain to the periphery through the NVU/PVU [125,128]. In combination with preclinical evidence on glymphatic–lymphatic functions [58,59,62], knowledge on NVU/PVU biology highlights the potential significance of cerebral vascular and perivascular tissue change and pathology in AD, as NVU/PVU alterations are likely associated with impaired brain metabolite clearance and hampered brain–brain and brain–periphery signaling, in addition to inefficient brain nutrient delivery [129,130,131]. Thus, NVU/PVU dysfunction may have regional and/or global brain effects, and may lead to generalized protein clearance failure and dysregulated brain–body communication.

### 4.2. Compound Proteinopathies in AD

**Figure 3:**
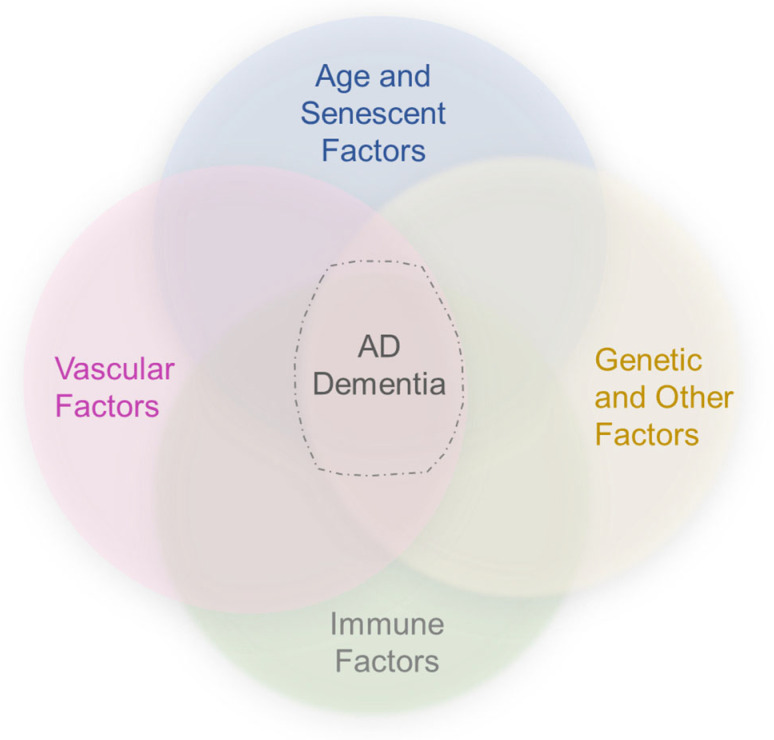
An updated framework suggests the convergence of vascular, immune, and other risk factors in AD. In combination with age, genetic and other factors, vascular and immune factors may converge to precipitate βA plaque and intraneuronal NFT formation, and induce other biological phenomena to cause dementia.

As highlighted in recent literature, significant subsets of AD dementia cases are attributable to TDP-43 inclusions and/or α-synuclein-positive Lewy bodies [1,132,133]. Studies show that non-βA and non-tau proteinopathies are not mutually exclusive of one another, nor with AD-NC [132,133]. In fact, cohort studies demonstrate that TDP-43 pathology combined with AD-NC accounts for 35–37% of pathology in elderly subjects with AD dementia [134,135], while Lewy body inclusions overall manifest in more than 50% [135,136]. Thus, common mechanisms may exist for proteinopathic diseases [1,137]. Increasing evidence supports the hypotheses that generalized protein misfolding phenomena in combination with impaired waste clearance efficiency and genetic predispositions may place persons at higher risk for dementing diseases (Figure 3) [44,54,137]. The stereotyped accumulation of protein inclusions from ventral to dorsal brain regions may further implicate perivascular glymphatic–lymphatic transport failure as a common driver of aberrant protein aggregate deposition or cognitive impairment [137].

### 4.3. Blood, Peripheral Signaling and Brain-Body Connections in AD

Intracranial vessels (i.e., lymphatic and blood vascular) and biofluids (i.e., ISF, CSF, and blood) convey innumerable signals, in addition to cytokines and chemokines discussed above. Secretory mechanisms at the BBB impart endocrine-like properties of BBB endothelium [138,139]. Moreover, neurons and neuroglial cells communicate with themselves, with one another, and with the periphery via receptors and extracellular molecules involving autocrine, paracrine, and endocrine signals that transmit in extracellular spaces [138,139,140]. Other serum-derived factors and biomolecules derived from brain and periphery are also transported through intracranial fluids [141,142,143,144,145,146,147]. Recent literature demonstrates that exosomes and ectosomes, small extracellular vesicles (EV) released by exocytosis and plasma membrane shedding, have critical effects on brain homeostasis [142,143,144,145,146]. Exosomes and ectosomes transport a diverse array of cargo including proteins, glycoproteins, lipids, nucleic acids, metabolites, and other biomolecules following their release into the microenvironment [142,143,144,145,146]. As they are capable of inducing phenotypic changes of cells upon fusion and uptake, they are predicted to participate in AD pathogenesis through various mechanisms and pathways [148].

Recent studies also indicate that microbiomes influence brain structure and function across aging [147]. Microbiota (i.e., bacteria, archae, viruses, protists and fungi) and host immune systems co-evolve across the lifespan and homeostasis of the brain–gut axis is reliant upon their efficient coupling and communication [147,148]. The host microbiome influences brain function and is increasingly recognized to modulate both peripheral and central immune responses, as pathogen-associated molecular patterns (PAMPs)/microbe-associated molecular patterns (MAMPs) trigger endogenous molecules such as damage-associated molecular patterns (DAMPs) that are recognized by tissue-resident immune cells via host pattern recognition receptors (PRRs), and cause cell activation while eliciting innate immune responses through a variety of mechanisms [147,148]. This includes increased production of cytokines, chemokines, neuropeptides, metabolic and other signals, as well as complement activation [147,148]. It has been shown that microbiota influence BBB permeability and infiltration of peripheral immune cells, including cytotoxic T-cells into brains of AD subjects, where they are associated with synaptic dysfunction and cognitive changes [147,148,149,150]. Imbalances of microbiome homeostasis impact peripheral immune cell trafficking and cause microglial activation within the brain, impacting brain function [147,148,149,150]. Likewise, brain–heart, brain–lung, and other brain–body connections are additionally influenced through bloodborne and immune factors that signal within the brain and at brain borders [150,151,152,153,154], and are subject to effects of the fluid and neuroimmune dysregulation [155].

## 5. Conclusions

Despite considerable advances in knowledge regarding brain structure and physiology and the dire need for new AD therapies, appropriate AD drug development strategies remain undefined. While aging, genetic and biochemical factors influence the formation of intracerebral βA and tau aggregates that constitute core AD-NC criteria, the bases for cognitive decline in persons with AD dementia are variable and complex. Together, the cerebral vasculature and brain lymphatic system supply the brain with oxygen and nutrients while removing waste metabolites and permitting transvascular and perivascular molecular exchange, signaling, and processing that are critical for brain health and homeostasis. These roles of the cerebral vascular–immune complex are fundamental to sensing and controlling the brain’s microenvironment.

A vascular–immune basis of disease, incorporating dysfunctional brain clearance mechanisms and signaling pathways, centered upon the NVU and PVU, may in part account for inconsistencies in AD pathology and grading (Figure 3). However, basic knowledge on the brain vascular and lymphatic structure and function in humans and their associations with AD remains incomplete. Further clinical investigation into biomarkers that serve as indicators of abnormal intracerebral fluid flow, immune dysfunction, and general protein transport efficiency in aging may yield a better understanding of appropriate therapeutic targets and etiologies involved in cognitive decline in persons afflicted with AD. While prior theories insufficiently explain AD pathobiology, recognition of a brain-wide fluid signaling and lymphatic drainage system raises novel concepts. Disorder of the brain’s vascular–immune system may encompass several previously proposed hypotheses of AD. Future priorities and challenges in understanding this system will include further defining healthy and diseased brain vasculature characteristics across the arteriovenous axis, age spectrum, and brain regions in humans and elucidating divergent effects of immune-related changes and vascular pathologies across comorbidities and stages of disease progression.

## Data Availability

Not applicable.
